# Mental health-related communication in a virtual community: text mining analysis of a digital exchange platform during the Covid-19 pandemic

**DOI:** 10.1186/s12888-022-04080-1

**Published:** 2022-06-25

**Authors:** C. Golz, D. Richter, N. Sprecher, C. Gurtner

**Affiliations:** 1 Department of Health Professions, Bern University of Applied Sciences, Murtenstrasse 10, 3008 Bern, Switzerland; 2 Center for Psychiatric Rehabilitation, Bern University Hospital for Mental Health, Bern, Switzerland

**Keywords:** Virtual communities, Sentiment analysis, Text mining, Covid-19, Mental health

## Abstract

**Background:**

Virtual communities played an important role in mental health and well-being during the Covid-19 pandemic by providing access to others and thereby preventing loneliness. The pandemic has accelerated the urge for digital solutions for people with pre-existing mental health problems. So far, it remains unclear how the people concerned communicate with each other and benefit from peer-to-peer support on a moderated digital platform.

**Objective:**

The aim of the project was to identify and describe the communication patterns and verbal expression of users on the inCLOUsiv platform during the first lockdown in 2020.

**Methods:**

Discussions in forums and live chats on inCLOUsiv were analysed using text mining, which included frequency, correlation, n-gram and sentiment analyses.

**Results:**

The communication behaviour of users on inCLOUsiv was benevolent and supportive; and 72% of the identified sentiments were positive. Users addressed the topics of ‘corona’, ‘anxiety’ and ‘crisis’ and shared coping strategies.

**Conclusions:**

The benevolent interaction between users on inCLOUsiv is in line with other virtual communities for Covid-19 and the potential for peer-to-peer support. Users can benefit from each other’s experiences and support each other. Virtual communities can be used as an adjuvant to existing therapy, particularly in times of reduced access to local health services.

## Introduction

The pandemic and measures imposed by the authorities to manage the spread of Covid-19 have led to an increase in loneliness, stress, anxiety and depression, a general decline in mental well-being and an increase in substance or alcohol abuse in the general population [1–3]. People with pre-existing mental health problems were at increased risk of poorer mental health outcomes due to reduced access to mental health services or measures such as quarantine or social isolation [4–7]. Lack of accessible mental health services might have led to people affected by the Covid-19 pandemic seeking alternative solutions for social inclusion. Especially during the first wave of the pandemic and the associated lockdown, individuals used digital solutions to maintain their connections with others by gathering in virtual communities (VC) [8, 9].

### Virtual communities

These VC differed regarding their aims, such as a VC for passive readers of daily Covid-19 infection reports or a VC with more active contributors through reciprocal emotional support and posting humorous content [9]. However, all VC had one common theme: they were initiated and moderated by the users [8, 9].

VC make an important contribution to the promotion and maintenance of mental health in contemporary society and promoting the processes of recovery [10]. As a result, they are firmly positioned in the range of available structures for health-related services. The advantages of VC are that they provide a forum for expressing and sharing service users’ opinions and experiences. Users with similar health-related problems and interests can exchange information in a protected environment or be in contact with each other regardless of time and location [11]. In VC users who are less likely to be in direct contact are more willing to share personal information [12]. In this context, exchanges with peers (i.e. persons with a comparable health-related problem) are particularly supportive in improving mental health [13], although only a few users are active in this way, with the majority only reading the shared experiences [14]. However, one disadvantage of VC is that unstructured, unmoderated peer-supported Internet offerings can also lead to an increase in the feeling of stress among users [15].

### inCLOUsiv

Low-threshold digital counselling and information services by health professionals during the Covid-19 pandemic have made a central contribution to the prevention of mental illness [16]. In this regard, a group of Swiss health researchers, communication specialists and mental health service users developed inCLOUsiv, which is a digital communication platform that combines the advantages of VC with a peer-led low-threshold counselling and information service provided by professionals. inCLOUsiv is meant to be accessible to all groups of people interested in the topic of mental health, but particularly addresses people with mental health issues. The platform provides information on how to manage, maintain and promote mental health, as well as giving users an opportunity for interaction and networking. Users are able to post and comment on articles and posts, and in addition they are able to join live discussions in which a specific topic is discussed with a moderator in a chat. The platform is operated by a Non-Governmental Organization specialized in the field of mental health and involves a team of communication specialists, health professionals and peer support workers.

inCLOUsiv combines the characteristics of a VC with structured inputs and moderation, therefore it cannot be assumed that users will interact in the same way as on user-led VC during the Covid-19 pandemic. Moreover, we could not find any other study describing the interaction among users on professional-led mental health-related VC during the pandemic. Therefore, this study aims to explore how users interact with each other on the inCLOUsiv platform during the first Covid-19-related lockdown. We further tried to answer the following questions:

- What Covid-19-related topics do the users exchange?
- To what extent is the exchange among the users benevolent?
- How and to what extent do users support each other?

## Methods

To answer the research question, we conducted a descriptive study with the natural language processing method, text mining (e.g. frequencies of words used or sentiment analysis) [8, 9].

For unstructured datasets such as text from VC, text mining is suitable because data preparation and analysis is automated and facilitates the identification of new information and relationships within comprehensive unstructured datasets [17].

### Ethics approval and consent to participate

This research was not covered by the Swiss Human Research Act, since no health-related personal data and user information was obtained (SR810.30, Article 2). Accordingly, no request to an ethics committee has been made. The study was conducted in accordance with the Declaration of Helsinki. Regarding informed consent, the users were informed about the study, voluntary nature of their participation and anonymization of extracted data in writing by e-mail, before completing registration on the platform and the Impressum of the platform. The users gave their informed consent by registering to the platform. Participants could withdraw from the study without giving a reason.

### Sample

We invited people with experience of mental illness and mental health professionals to register on the platform. The platform was promoted to the target groups via newsletters, announcements on the partners’ websites and social media channels (LinkedIn, Facebook, etc.). The invitation was sent by e-mail to the researchers’ network and national established peer groups, such as Peerplus, Network Recovery, Experienced Involvement Switzerland, and others. The e-mail included information about the platform inCLOUsiv as well as information about the study. However, other interested people were also able to register on the platform. People were eligible to participate actively, if they had access to the internet and an own e-mail address. Registered users interacted with others in the forums, live discussions and posts, or they were able to access the contents (e.g. articles) without registration (read-only). Users were able to log onto the platform with an e-mail address, a name and a password of their own creation, to exchange ideas with other users and with the editorial team and moderators.

### Data collection

Data collection took place over the period 24 March–17 May 2020. Data from the forums and live discussions were stored in the MySQL database of inCLOUsiv. The raw data were exported without profile information (name and e-mail address) after the test phase was completed.

### Data analysis

Preparation and analysis of the data were carried out in the statistical program R 3.6.1 [18] based on tidy data principles [19] with the primary ‘tm’ packages [20] and ‘tidytext’ [19]. The dataset was prepared for analysis in several steps. (1) All the words were identified as individual elements in a corpus (this process is referred to as tokenization) and, subsequently, the distribution of the words in the corpus was calculated. (2) On this foundation, all stop words, numbers, punctuation marks and other words not relevant for the analysis (such as names, greetings) were deleted, so that the starting point for the analysis is an anonymized dataset consisting only of the core contents of the conversations. (3) The words were reduced to their dictionary root or base form (this process is known as lemmatization) using spaCyr packages [21] with the German-language-specific package ‘de_core_new_lg’. This was important for the analysis, so that word forms with the same root, such as *‘makes’* [macht], *‘made’* [gemacht] and *‘make’* [mache], are aggregated in the basic form *‘to make’* [machen]. (4) On the basis of the pre-processed corpus, frequencies, correlations (phi coefficient), n-gram and sentiment analyses were carried out. An n-gram is a sequence of *n* elements from a given text: for example, the bigram (where *n* = 2) comprises a sequence of two words. For sentiment analysis, the German-language-specific resource SentimentWortschatz (SentiWS) [22] was used. In its current version, SentiWS consists of 1650 positive basic word forms and 1800 negative basic word forms, with an overall assigned polarity from − 1 (strongly negative) to 1 (strongly positive). For example, the word ‘great’ [super] has a positive polarity with a value of 0.5012 and the word ‘bad’ [schlecht] has a negative polarity with a value of − 0.7706.

## Results

After the platform was publicly accessible, the number of registered users as well as daily unregistered visitors increased continuously. By 4 May 2020, almost 700 registered users were recorded. At the beginning of April there were up to 10,000 page views per day. However, the amount of website visits fell sharply during April. In the last week of the evaluation (28 April–4 May 2020), an average of 795 views per day were recorded (min. 62; max. 1159). An assessment by username and activity also revealed that for this portion of 2300 visits, the majority were passive consumers of the content and did not participate in the dialogue. As registration only required a valid e-mail address and a name, no further assertions can be made about the users. The analysis was based on an adjusted dataset of 31,764 words.

### Frequencies, n-grams and correlations

**Fig. 1:**
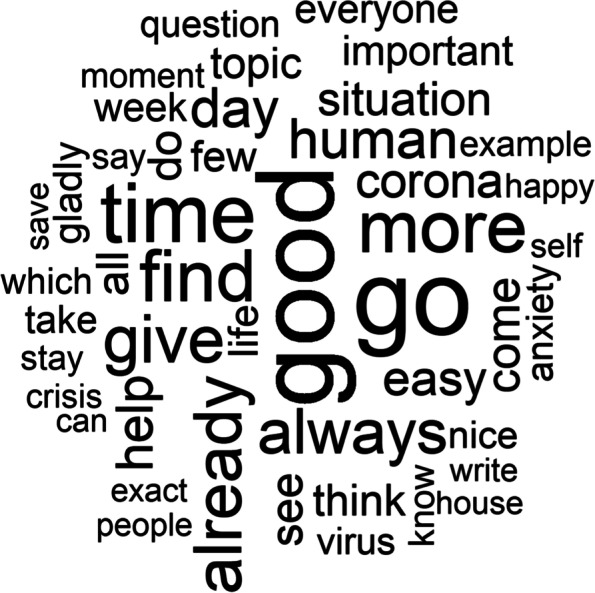
Wordcloud containing the most frequently used words (*n* > 75)

Figure 1 shows the most frequently used words (*n* > 75). The most widely used reference word was *‘good’* (*n* = 396), even though in the preparation, greetings (e.g. good morning) and sign-off words (e.g. goodbye) were removed. Topic-specific words such as *‘corona’* (*n* = 144) and *‘virus’* (*n* = 100) are apparent. The words *‘crisis’* (*n* = 82) and *‘anxiety’* (*n* = 98) give an initial indication of possible discussion points in the forums and live chats.

**Fig. 2:**
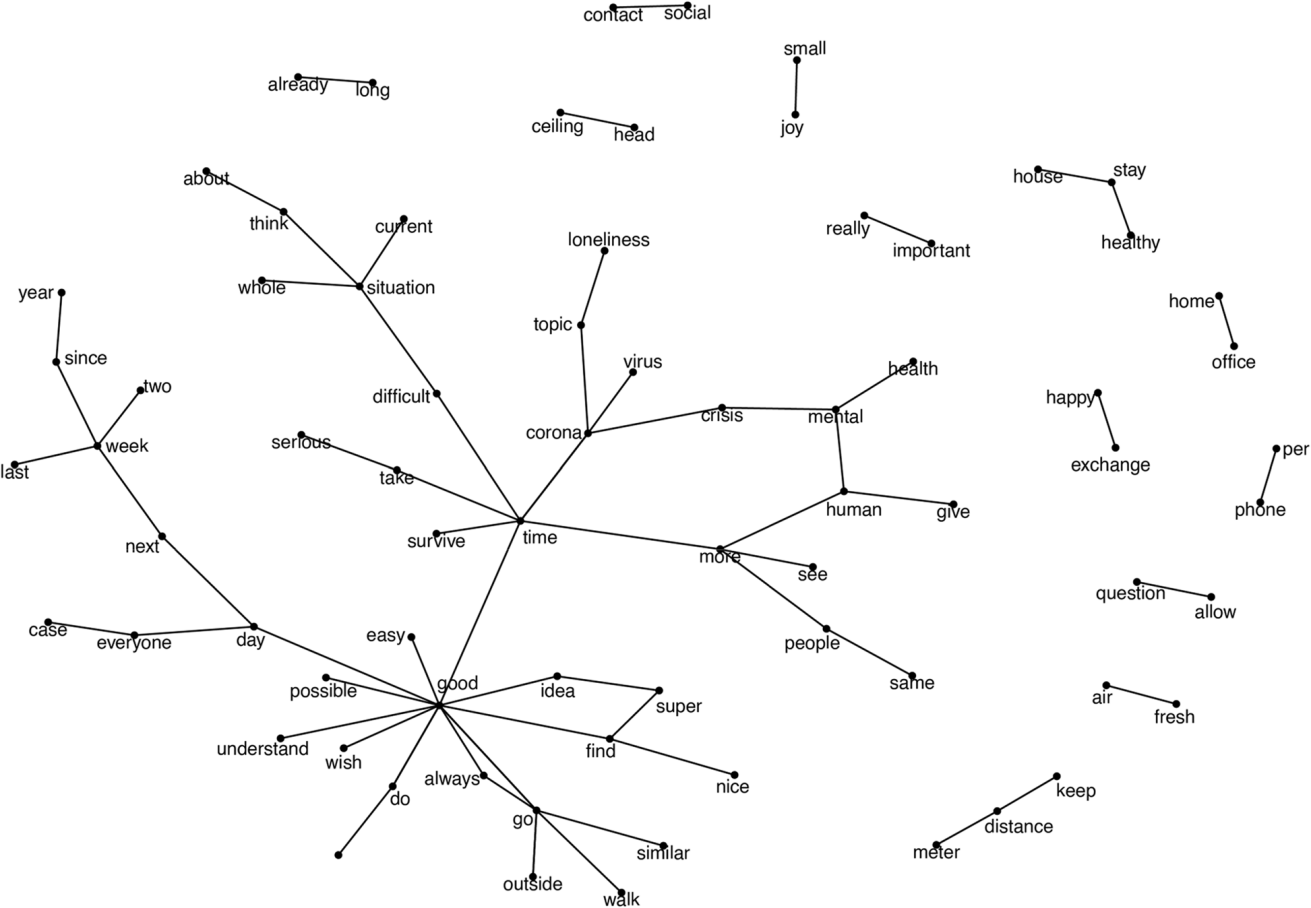
Bigram visualization in the forums and live discussions (*n* > 5)

Analysis of the bigrams (Fig. 2), filtered according to a frequency of *n* > 5, showed several clusters, the majority of which consisted of two combined constructs, such as the topics prevailing at that time in the media: for example, *‘keep’ ‘distance’* or *‘home’ ‘office’*. The construct *‘corona’* was a nodal point that was associated, on the one hand, with stressing topics and, on the other hand, with a cluster consisting of supportive (i.e. empathetic) content. The participants exchanged views on the theme of *‘loneliness’* and the impact of the *‘crisis’* on *‘mental health*’. *‘Time’* also stands as a nodal point between stressing and supportive topics. Thus, *‘time’* was experienced during the test phase as a *‘difficult’ ‘situation’* that needs to be *‘taken’ ‘seriously’*. Also shared among the participants were tips for coping strategies, how to *‘take’ ‘time’*, to *‘do yourself some good’* or *‘go outside’* and *‘go for a walk’*.

In addition to visualization of the bigrams, correlation analysis (phi coefficient) shows that some topics often come up together but do not directly follow each other in sequence, as is the prerequisite for analysis of the n-grams (Table 1). Here, too, the challenges faced by participants during the first wave of Covid-19 show up as *‘anxiety’* due to the *‘pandemic’* or the *‘virus’*.

**Table 1:** Correlations of second words

Word 1	Word 2	Phi coefficient
mental	health	0.377
full	energy	0.362
relaxation	sports	0.328
corona	virus	0.310
voice	hear	0.272
pandemic	anxiety	0.261
last	strength	0.264
virus	anxiety	0.260
discussion	look forward	0.255
few	tips	0.236

On the other hand, there are also possible coping strategies, such as *‘relaxation’* and *‘sports’* or *‘listening’* and *‘hearing a voice’*, which in this context is not discussed as a symptom,but as a coping strategy.

The analysis according to bigrams and trigrams, with a focus on advice on coping strategies with the words *‘idea’* or *‘tip’*, without narrowing down by frequency, revealed a variety of activities, such as sports (e.g. walking, jogging), games (e.g. board games, Lego), writing (e.g. letters, diary), reading and talking with relatives and affected people (e.g. forum, phone calls, WhatsApp) who were recommended to each other.

### Sentiment analyses

Most of the words did not describe neutral sentiments. The results show a low tendency for positive sentiment between users, with a polarity ranging between negative (− 1) and positive (+ 1) with an average value of 0.04, in which words with a positive classification were represented with a preponderance of 72% (3737 vs. 1425). Table 2 summarizes the 15 most commonly used positive and negative words. The word *‘good’* (*n* = 396) with positive polarization was most frequently used, followed by *‘help’* (*n* = 156). The most frequently used word with a negative polarization was *‘anxiety’* (*n* = 98), followed by *‘crisis’* (*n* = 82).

**Table 2:** Sentiment analysis with the 15 most common feelings

Negative	***N*** (%)	Weighting*	Positive	***N*** (%)	Weighting*
anxiety	98 (7)	− 0.514	good	396 (10)	0.372
crisis	82 (6)	−0.362	help	156 (4)	0.373
difficult	64 (4)	−0.025	simple	150 (4)	0.004
unfortunately	53 (4)	−0.479	important	116 (3)	0.382
problem	45 (3)	−0.387	look forward	82 (2)	0.220
small	44 (3)	−0.272	secure	78 (2)	0.004
short	30 (2)	−0.001	exactly	78 (2)	0.004
challenge	28 (2)	−0.001	experience	73 (2)	0.004
difficult	27 (2)	−0.001	current	60 (2)	0.004
resist	26 (2)	−0.001	understand	56 (1)	0.096
bad	26 (2)	−0.771	new	56 (1)	0.004
old	24 (2)	−0.001	know	54 (1)	0.004
lack	22 (2)	−0.537	hope	54 (1)	0.232
worry	21 (1)	−0.362	super	53 (1)	0.501
loneliness	21 (1)	−0.339	learn	42 (1)	0.250

*Weighting calculated as Pointwise Mutual Information [15]

**Fig. 3:**
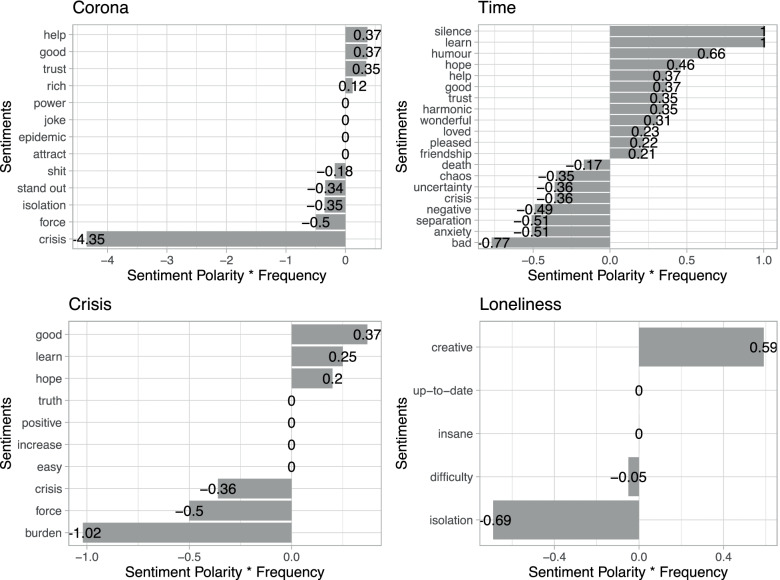
Sentiment analysis with preceding words

An in-depth sentiment analysis focusing on thematic words such as *‘loneliness’*, *‘crisis’*, *‘corona’* and *‘time’* could show that every word, whether it appears to have a negative or positive connotation, carries both polarities (see Fig. 3). For example, the word *‘loneliness’* is related to *‘isolation’* and also to *‘being creative’*. As the polarity of sentiments can range from − 1 to 1, it is apparent that relatively strong words with negative polarization were used in comparison with words with a positive polarization.

## Discussion

The aim of the study was to describe the users’ interaction on the platform inCLOUsiv during the first lockdown of the Covid-19 pandemic. The findings show that the users’ communication behaviour was well meaning and supportive. The positive and supportive interaction seems to go in line with other mental health-related communication in VC [8, 9]. Thus, the way in which users interact with each other does not differ between user-led VC and VC managed by organizations or professionals. This benevolent communication represents an important basis for a successful VC, as it motivates and thus stimulates further exchange [23]. Furthermore, the benevolent exchange among people with mental health issues seems to be a necessary basis for the development of coping strategies [24], which may indicate that professional-led VC can be used as an adjuvant to existing treatment.

The identified topics of *‘anxiety’* and ‘*loneliness’* in connection with the virus and isolation go hand in hand with the current literature: that the degree of anxiety has increased among the population [25, 26]. However, current literature on the effect of VC on users’ health status [27] or mental health during the Covid-19 pandemic [8] is sparse. Further research is needed to understand and describe the effect of such VC on the users’ health status as well as their well-being, but this is a challenge because, from the perspective of the users, the advantage of a VC and the support it provides is anonymity [28]. Online platforms seem to be particularly relevant for younger people with mental health problems. The focus is on anonymity, sharing experiences or easy and unlimited accessibility [28]. In this study, no information was collected on the demographic data of the participants, which makes it impossible to sub-analyse by group.

Another important contribution to the sustainable success of such VCs seems to be the active participation of health professionals in the exchange, because the absence of these representatives could raise scepticism towards them [29]. In this study, health professionals were encouraged to participate actively, but their involvement was limited to the content of the live discussions, in which users had the opportunity to engage with both peer support workers and professionals. How health professionals can be further motivated to participate actively in VC should be the subject of further research.

For longer-term success, it appears to be important to promote the development of cohesion [30]. The chosen participatory approach on the inCLOUsiv platform and the possibility of introducing relevant topics could indicate development towards a community, at least for the phase described. Cultural circumstances constitute an important foundation for this sustainable added value [30]. Eichenberg and Brähler [31] report a high level of acceptance of virtual support offerings among the population but point out that these are not yet used as much in standard health care offerings as is the case in everyday life. inCLOUsiv contributes to closing this gap and provides a virtual offering for the population that is used in everyday life and can provide an interface to the standard health care offerings. Users can access the platform with flexibility in terms of time and location. The low-threshold offer also seems to have attracted a great deal of interest, as access to inCLOUsiv was passive (i.e. used only for content consumption) in the majority of cases.

## Limitations

The innovative approach to analysis of the discussion content provides insights into the main topics during the Covid-19 pandemic and also the communication between users. The results were able to demonstrate the topics that people with mental illness are dealing with during the pandemic, which coping strategies they experience as helpful and also that they consequently exchange with each other on inCLOUsiv.

One weakness of the study is the short survey period of 3 months, which generated a dataset that did not allow for sub-analysis of sentiments by forum or live discussion. In addition, the sampling procedure may have resulted in the sole voluntary participation of users who were already engaged and not overburdened. Furthermore, the proportion of active users was small in relation to the total number of visits. However, this is in line with another study about user activity in Covid-specific VC [9]. While health professionals were also asked about active participation on the platform, this group seemed restrained and more present as moderators or participants in the announced live discussions. Due to a lack of personal and health-related information on users, no differentiating analyses could be made. However, this was planned to ensure anonymity and low-threshold barriers for registration on the platform, which is particularly important for people with mental health problems [28]. In the first wave of Covid-19, mental health systems were not adequately prepared in terms of suitable services or reduced access to services [7], therefore Covid-specific VC might beneficially compensate for some aspects of mental health services for people with a mental illness. Thus, decision makers in the mental health system should consider expanding their services with VC as there was a demonstrable need for low-threshold exchange on mental health during the Covid-19 pandemic [8, 9, 32].

## Conclusions

The results indicate that users have benevolent discussions and support each other in managing their mental health during the pandemic on professional-led VC. Users state that they benefit from the experience of others and exchange useful coping strategies among themselves. The benevolent communication among the users suggests that the users consciously communicate supportively and that there is no stigma or bullying among them. For health professionals, this means that such low-threshold support offerings can be used as an adjuvant to existing therapy, particularly in times of reduced access to local health services.

For research, the analysis method can be used after developing the script for regular evaluation of content and sentiments on VC. Data from longitudinal studies can provide an in-depth insight into the course of the users’ sentiments towards mental health-related topics.

## Data Availability

The dataset generated and analysed during the current study is publicly available on the OSF platform as a .txt file: www.osf.io/hr5ak.

## Abbreviations

VC: Virtual communities
